# NECTIN4 Expression in Extramammary Paget’s Disease: Implication of a New Therapeutic Target

**DOI:** 10.3390/ijms21165891

**Published:** 2020-08-16

**Authors:** Maho Murata, Takamichi Ito, Yuka Tanaka, Yumiko Kaku-Ito, Masutaka Furue

**Affiliations:** 1 Department of Dermatology, Graduate School of Medical Sciences, Kyushu University, Fukuoka 812-8582, Japan; muratama@dermatol.med.kyushu-u.ac.jp (M.M.); yukat53@med.kyushu-u.ac.jp (Y.T.); kyumiko@dermatol.med.kyushu-u.ac.jp (Y.K.-I.); furue@dermatol.med.kyushu-u.ac.jp (M.F.); 2 Research and Clinical Center for Yusho and Dioxin, Kyushu University Hospital, Fukuoka 812-8582, Japan; 3 Division of Skin Surface Sensing, Department of Dermatology, Faculty of Medical Sciences, Kyushu University, Fukuoka 812-8582, Japan

**Keywords:** extramammary Paget’s disease, NECTIN4, enfortumab vedotin

## Abstract

Extramammary Paget’s disease (EMPD) is a rare skin cancer arising in the anogenital area. Most EMPD tumors remain dormant as in situ lesions, but the outcomes of patients with metastatic EMPD are poor because of the lack of effective systemic therapies. Nectin cell adhesion molecule 4 (NECTIN4) has attracted attention as a potential therapeutic target for some cancers. Urothelial cancer is one such cancer, and clinical trials of enfortumab vedotin, a drug-conjugated anti-NECTIN4 antibody, are ongoing. However, little is known regarding the role of NECTIN4 in EMPD. In this study, we conducted immunohistochemical analysis of NECTIN4 expression in 110 clinical EMPD samples and normal skin tissue. In normal skin, positive signals were observed in epidermal keratinocytes (particularly in the lower part of the epidermis), eccrine and apocrine sweat glands, inner and outer root sheaths, and matrix of the hair follicles. The most EMPD lesions exhibited strong NECTIN4 expression, and high NECTIN4 expression was significantly associated with increased tumor thickness, advanced TNM stage, and worse disease-specific survival. These results support the potential use of NECTIN4-targeted therapy for EMPD. Our report contributes to the better understanding of the pathobiology of NECTIN4 in the skin and the skin-related adverse effects of NECTIN4-targeted therapy.

## 1. Introduction

Extramammary Paget’s disease (EMPD), first reported by Crocker in 1889, is an uncommon skin cancer that mainly affects apocrine sweat gland-rich areas [1,2]. Extramammary Paget’s disease most frequently arises in the anogenital area in elderly people and less commonly in the axillary area [2,3,4]. This disease typically affects Caucasian woman and Asian men older than 60 years [3,4,5,6]. Most EMPD tumors remain restricted to the epidermis as in situ lesions, and they carry a good prognosis because of their slow-growing nature [1,7]. However, approximately 15–40% of EMPD lesions display dermal invasion, increasing the risk of lymph node and distant metastases [2,4]. Complete surgical removal is the first-line treatment for EMPD in the localized stage, but it is sometimes difficult to determine the appropriate resection line because of the unclear tumor border of EMPD, leading to incomplete excision and subsequent tumor progression to metastasis [8,9,10,11]. Although several therapeutic modalities such as radiation therapy, chemotherapy, and molecular targeted therapy have been reported, their efficacy in treating unresectable EMPD is unsatisfactory. Thus, new treatment modalities are required [4,12,13,14].

In recent years, nectin cell adhesion molecule 4 (NECTIN4) has been identified as an important factor in several malignant tumors, including urothelial carcinoma, gastric cancer, thyroid cancer, breast cancer, esophageal cancer, and ovarian cancer [15,16,17,18,19,20,21]. Nectin cell adhesion molecule 4 is a member of the NECTIN family of immunoglobulin-like adhesion molecules [22,23]. It has an important role in mediating cell-cell adhesion via the organization of epithelial and endothelial junctions [18,23]. Moreover, NECTIN4 is the epithelial receptor for measles virus [24,25]. Nectin cell adhesion molecule 4 is normally expressed in the epithelium of the bladder, epidermis of the skin, salivary glands (ducts), and mammary glands [16,22]. Interestingly, NECTIN4 is overexpressed in some epithelial cancers, and its overexpression is correlated with tumor progression and patient outcome [16,22,23,26]. However, the detailed expression of NECTIN4 in human skin and its role in skin tumors have not been examined. In this study, we first investigated the distribution and characteristics of NECTIN4 expression in normal skin and skin appendages. We then examined NECTIN4 expression in 110 clinical EMPD samples using immunohistochemistry, and the associations between NECTIN4 expression and patient survival were assessed.

## 2. Results

### 2.1. Characteristics of the Study Cohort

The comprehensive demographic and clinical data of all 110 patients with primary EMPD are shown in Table 1. The mean patient age was 73.7 years (range: 47–91). In total, 69 patients (62.7%) were male, and the remaining 41 patients (37.3%) were female. The most common primary tumor site was the anogenital area (94.5%) followed by the axillae (5.5%). The TNM stage was defined in accordance with an EMPD-specific staging system proposed by Ohara et al. in 2016 [7]. The majority of the patients (85.5%) presented with TNM stage I or II lesions (no lymph node or distant metastasis), whereas 14.5% of patients had stage III or IV disease (presence of at least one lymph node or distant metastatic lesion). The tumor thickness (TT) in the patient cohort was as follows: TT ≤ 1 mm in 69.1% of patients; 1 < TT ≤ 2 mm in 9.1% of patients; 2 < TT ≤ 4 mm in 4.5% of patients; and TT > 4 mm in 6.4% of patients.

### 2.2. NECTIN4 Expression in Normal Skin and Extramammary Paget’s Disease (EMPD)

**Figure 1:**
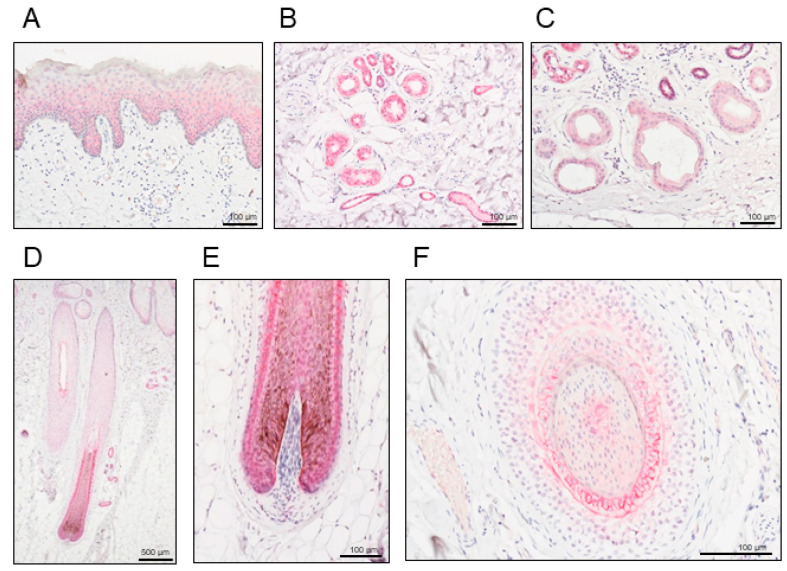
Representative histopathological images of nectin cell adhesion molecule 4 (NECTIN4) staining in human normal skin. Positive signals are presented in red. (**A**) Epidermis, (**B**) eccrine sweat glands, (**C**) apocrine sweat glands, and (**D**–**F**) inner and outer root sheaths, and matrix of the hair follicles. Scale bars: 100–500 μm.

We first examined the detailed distribution of NECTIN4 in normal human skin tissues. Representative histopathological images of NECTIN4 staining in human normal skin are presented in Figure 1. Positive NECTIN4 staining was indicated by a red color. Epidermal keratinocytes (Figure 1A), eccrine sweat glands (Figure 1B), apocrine sweat glands (Figure 1C), the inner and outer root sheaths, and matrix of the hair follicles (Figure 1D–F) had positive signals for NECTIN4. In particular, the eccrine sweat glands exhibited strong signals (Figure 1B).

**Figure 2:**
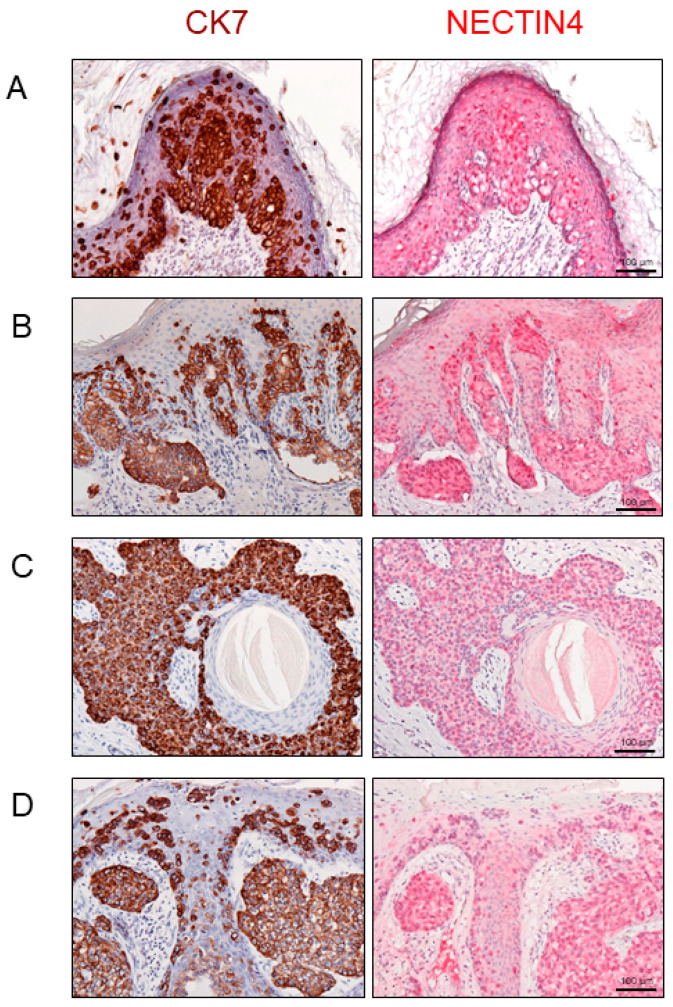
(**A**–**D**) Representative histopathological images of cytokeratin 7 (CK7; brown, left) and nectin cell adhesion molecule 4 (NECTIN4; red, right) staining in extramammary Paget’s disease. H-score for NECTIN4 were: (**A**) 111, (**B**) 210, (**C**) 200, (**D**) 130. Scale bars: 100 μm.

We then examined NECTIN4 expression in EMPD tissue samples. Moreover, cytokeratin 7 (CK7) was stained to clearly delineate the EMPD tumor cells. Positive CK7 staining was indicated by a brown color. Representative images of NECTIN4 or CK7 staining in EMPD lesions are presented in Figure 2A–D. Positive NECTIN4 staining was mainly found in the cytoplasm and on the membranes of tumor cells [20]. Supplementary Figure S2 summarizes the H-score for NECTIN4 in 110 EMPD tissues [22,27]. The mean H-score was 124.02, and the median score was 110 (range: 19–275). We divided the samples into two groups based on the mean H-score: NECTIN4-low (H-score ≤ 124) and NECTIN4-high (H-score > 124).

### 2.3. Association between NECTIN4 and Clinicopathological Factors in EMPD

Table 2 presents the associations between immunohistochemical NECTIN4 expression and clinicopathological factors. TT was categorized as ≤1 mm or >1 mm in accordance with previous reports [4,28]. In total, 41 (37.3%) and 69 samples (62.7%) were categorized into the NECTIN4-high and NECTIN4-low groups, respectively. Among these factors, male sex (odds ratio 3.08, 95% confidence interval (CI) 1.24–7.08; *p* = 0.014), TNM advanced stage (odds ratio 3.67, 95% CI 1.27–9.38; *p* = 0.017), and thicker TT (odds ratio 3.60, 95% CI 1.31–9.10; *p* = 0.017) were significantly associated with high NECTIN4 expression.

### 2.4. Prognostic Impact of NECTIN4 Expression

**Figure 3:**
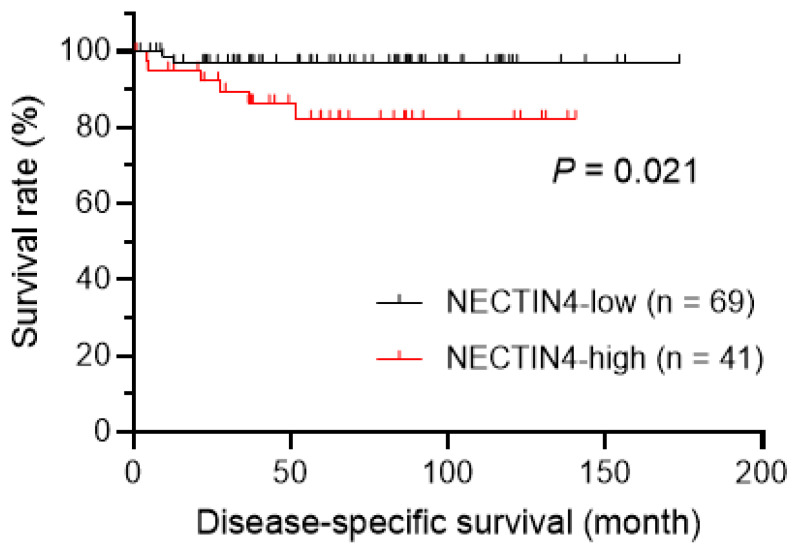
The correlation of nectin cell adhesion molecule 4 (NECTIN4) expression with disease-specific survival (DSS) in patients with extramammary Paget’s disease was determined using the Kaplan–Meier method and log-rank test. NECTIN4-high patients had significantly shorter DSS than NECTIN4-low patients. *p* < 0.05 indicated a statistically significant difference.

Next, we assessed the prognostic impact of NECTIN4 expression in EMPD. As shown in Figure 3, patients with NECTIN4-high EMPD had significantly shorter disease-specific survival (DSS) than those with NECTIN4-low EMPD (hazard ratio 0.19, 95% CI 0.044–0.79; *p* = 0.021). In multivariate analysis, none of the factors included in the model (age, sex, TT, TNM stage, and NECTIN4 expression) were significantly associated with DSS (Table 3). 

## 3. Discussion

The NECTIN family comprises Ca^2+^-independent immunoglobulin-like cell adhesion molecules and includes four members (NECTIN1–4) [22,23]. They are involved in several functional processes, including cell adhesion, movement, proliferation, differentiation, and polarization [17,18]. Nectin cell adhesion molecule 4, also known as poliovirus receptor-related protein 4, is involved in the formation and maintenance of adherens junctions in cooperation with cadherins, and it is also a receptor for measles virus, mediating its endocytosis [24,25]. Nectin cell adhesion molecule 4 is expressed during fetal development, and its expression is attenuated in adulthood, contrary to the extensive expression of other nectins in adult tissue [29]. Low NECTIN4 expression has been reported in various adult normal tissues [20,22]. The skin is a highly sophisticated tissue that protects the body against continuous external injuries. Although NECTIN4 expression has been demonstrated in human normal skin, we for the first time examined its detailed expression in the skin and skin appendages. In this study, we found ubiquitous expression of NECTIN4 in keratinocytes, particularly in the lower part of the epidermis, in which epidermal cells with proliferative ability reside. Interestingly, strong NECTIN4 expression was observed in the outer and inner root sheaths, and matrix of the hair follicles and sweat gland epithelium. These findings support the potential efficacy of recently developed NECTIN4-targeted therapies for malignant tumors derived from skin epidermis and skin appendages. This detailed localization of NECTIN4 should improve the understanding of the adverse effects of target therapies on the skin.

Importantly, most EMPD lesions exhibited strong NECTIN4 expression. Although the origin of EMPD is unknown, some researchers have described a correlation between EMPD and apocrine glands because EMPD arises in apocrine-rich areas. In the current study, normal human apocrine glands displayed weak but definite NECTIN4 expression. Conversely, NECTIN4 expression in EMPD was stronger than that in apocrine glands and the epidermal keratinocytes that we used as an internal positive control for NECTIN4. In general, most EMPD tumors grow slowly, and they can be cured when complete surgical removal is achieved in the localized stage. However, patient outcomes are poor once EMPD metastasizes [4]. For metastatic EMPD, several anticancer agents have been attempted including taxanes, platinum-containing drugs, 5-fluorouracil, epirubicin, vincristine, and mitomycin C. However, no standard chemotherapeutic regimen has been established, and the treatment results have been unsatisfactory [4]. New treatment options are therefore required.

It is interesting that high NECTIN4 expression in EMPD is correlated with TT, which is a recently identified prognostic factor, and advanced TNM stage [7,28]. High NECTIN4 expression was also significantly correlated with worse DSS. These findings suggest the NECTIN4-targeted therapy should be more effective against advanced EMPD. As a NECTIN4-targeted therapy, enfortumab vedotin has recently entered clinical use [15,16,30]. Enfortumab vedotin is an antibody-drug conjugate composed of an anti-NECTIN4 antibody bound to monomethyl auristatin E (MMAE), an antimitotic agent that inhibits microtubule assembly. Enfortumab vedotin binds to cells expressing NECTIN4 with high affinity, thereby triggering the internalization and release of MMAE in target cells and leading to cell cycle arrest and apoptosis [30]. A phase II trial of patients with urothelial carcinoma, which highly expresses NECTIN4, has been reported [30]. In this single-arm study, patients who previously received platinum chemotherapy and anti-PD-1/L1 therapy were treated with intravenous enfortumab vedotin administration. The response rate was meaningful, with a confirmed objective response rate of 44%, including complete responses in 12% of patients [30]. Some phase I studies have reported a manageable and tolerable safety profile for the drug [15,16,30]. A confirmatory phase III study is ongoing [30,31]. 

In summary, we demonstrated the detailed expression of NECTIN4 in normal skin and skin appendages. Most EMPD tumors exhibited high NECTIN4 expression, which was significantly correlated with advanced TT and TNM stage. NECTIN4-targeted therapies, such as enfortumab vedotin, could be new treatment options for unresectable EMPD.

## 4. Materials and Methods 

### 4.1. Patients

This retrospective review of our patients was conducted according to the guidelines of the Declaration of Helsinki. This study was approved by the Ethics Committee of Kyushu University Hospital (Nos. 26-1 and 30-363). We retrieved data for 110 patients with primary EMPD lesions who were treated at the Department of Dermatology, Kyushu University (Fukuoka, Japan) between January 1997 and December 2018. At least three experienced dermatopathologists confirmed the diagnosis. Secondary EMPD, which involved direct invasion from the visceral organs, was carefully excluded. The clinical and demographic data of all patients were collected from patients’ files and analyzed. 

### 4.2. Immunohistochemistry

We examined 110 EMPD skin samples, and normal skin samples from genital and scalp region. All formalin-fixed (24 h in 10% buffered formalin), paraffin-embedded tissues were obtained from the archives of our hospital. Immunohistochemical staining was performed as reported previously [32,33]. Briefly, tissue samples were cut into 4 μm sections. For NECTIN4, antigen was retrieved using Heat Processor Solution pH 9 (Nichirei Biosciences, Tokyo, Japan) at 100 °C for 40 min. The primary antibody was diluted with Dako REAL Antibody Diluent (s2022; Dako Denmark A/S, Glostrup, Denmark). The sections were incubated with rabbit anti-human NECTIN4 (1:150, ab192033; Abcam, Cambridge, UK) as the primary antibody for 30 min at room temperature followed by incubation with N-Histofine Simple Stain AP MULTI (414261; Nichirei Biosciences) as the secondary antibody for 30 min at room temperature. Immunoreactions were detected using FastRed II (415261; Nichirei Biosciences) as a chromogen, and specimens were counterstained using hematoxylin. For CK7, antigen was retrieved via incubation with protease (715231; Nichirei Biosciences) for 5 min followed by mouse anti-human CK7 (prediluted by supplier, 713481; Nichirei Biosciences) as the primary antibody for 30 min at room temperature. We then incubated sections with N-Histofine Simple Stain MAX-PO MULTI (724152; Nichirei Biosciences) as the secondary antibody for 30 min at room temperature. We detected immunoreactions using 3,3′-diaminobenzidine tetrahydrochloride (725191; Nichirei Biosciences) as a chromogenic substrate followed by counterstaining with hematoxylin.

### 4.3. Evaluation of NECTIN4 Immunohistochemical Staining

The immunohistochemical results were evaluated by a semiquantitative approach using the H-score [22,27]. The intensity of staining was graded as follows: no staining (0), weakly positive (1+), moderately positive (2+), and strongly positive (3+). The epidermis was used as an internal control, and its score was 2+ (Supplementary Figure S1). The staining of NECTIN4 in EMPD was generally homogeneous. The H-score of NECTIN4 was calculated as the percentage of positive cells (0–100%) multiplied by the staining intensity (0–3+), with the final score ranging from 0 to 300. The H-score was calculated by counting tumor cells in three random high-power fields (×200). Two independent dermatologists (M.M. and T.I.) who were blinded to the clinical information assessed the sections. Images were taken using an ECLIPSE 80i microscope (Nikon, Tokyo, Japan). 

### 4.4. Statistical Analysis

All statistical analyses were performed using GraphPad Prism version 8.3 (GraphPad Software, San Diego, CA, USA) and JMP version 14.2 (SAS Institute, Cary, NC, USA). To analyze the relationship between two variables, Fisher’s exact test was used. DSS was calculated using the Kaplan–Meier method and the log-rank test. For multivariate survival analysis, we used the multivariate Cox proportional hazards regression model. *p* < 0.05 indicated a statistically significant difference.

## Figures and Tables

**Table 1:** Basic demographic and clinical characteristic data of all 110 patients with primary extramammary Paget’s disease

Parameters	
Age, years	
Average	73.7
Median	73.0
Range	47–91
Sex	
Male	69 (62.7%)
Female	41 (37.3%)
Tumor site	
Anogenital area	104 (94.5%)
Others	6 (5.5%)
TNM stage	
I–II	94 (85.5%)
III–IV	16 (14.5%)
Tumor thickness (TT)	
TT ≤ 1 mm	76 (69.1%)
1 < TT ≤ 2 mm	10 (9.1%)
2 < TT ≤ 4 mm	5 (4.5%)
TT > 4 mm	7 (6.4%)
Unknown	12 (10.9%)

Data are presented as *n* (%) unless otherwise indicated.

**Table 2:** Factors associated with nectin cell adhesion molecule 4 (NECTIN4) expression.

	NECTIN4 Expression		
Parameters	Low	High	*p*-Value
Age (years)			
<70	19 (17.3%)	17 (15.5%)	0.15
≥70	50 (45.5%)	24 (21.8%)	
Sex			
Male	37 (33.6%)	32 (29.1%)	0.014
Female	32 (29.1%)	9 (8.2%)	
Tumor site			
Anogenital area	63 (57.3%)	41 (37.3%)	0.082
Others	6 (5.5%)	0 (0%)	
TNM stage			
I–II	62 (56.4%)	29 (26.4%)	0.017
III–IV	7 (6.4%)	12 (10.9%)	
Tumor thickness			
≤1 mm	57 (51.8%)	19 (17.3%)	0.017
>1 mm	10 (9.1%)	12 (10.9%)	
Unknown	2 (1.8%)	10 (9.1%)	
**Total**	69 (62.7%)	41 (37.3%)	

Data are presented as n (%). *p* < 0.05 indicated a statistically significant difference.

**Table 3:** Factors associated with disease-specific survival in Cox multivariate analysis

Variable	Hazard Ratio	95% CI	*p*-Value
Age, years	0.80	0.58–0.96	0.061
Sex, male	0.47	0.075–2.94	0.42
TNM stage, III or IV	3.01 × 10^12^	0	0.99
TT, >1 mm	7.02	0.24–200.58	0.25
NECTIN4 expression, high	0.07	0.0018–2.39	0.14

Abbreviations: TT, tumor thickness; CI, confidence interval; NECTIN4, nectin cell adhesion molecule 4. *p* < 0.05 indicated a statistically significant difference.

## Supplementary Materials

Supplementary Materials can be found at https://www.mdpi.com/1422-0067/21/16/5891/s1. Figure S1: Staining intensity of the nectin cell adhesion molecule 4 (NECTIN4) antibody. Positive signals are indicated by red. (A) Negative staining (0), (B) weakly positive staining (1+), (C) moderately positive staining (2+), and (D) strongly positive staining (3+). Figure S2: H-score for nectin cell adhesion molecule 4 (NECTIN4) staining in extramammary Paget’s disease.

## Abbreviations

EMPD	extramammary Paget’s disease
NECTIN4	nectin cell adhesion molecule 4
CK7	cytokeratin 7
DSS	disease-specific survival
TT	tumor thickness
95% CI	95% confidence interval
