# Supplementary material for: NECTIN4 Expression in Extramammary Paget’s Disease: Implication of a New Therapeutic Target
Source: Int J Mol Sci. 2020 Aug 16;21(16):5891. doi: 10.3390/ijms21165891 (PMC7460664; doi:10.3390/ijms21165891)
Supplement: Supplementary file 1 [file ijms-21-05891-s001.pdf]

Supplementary Figure S1

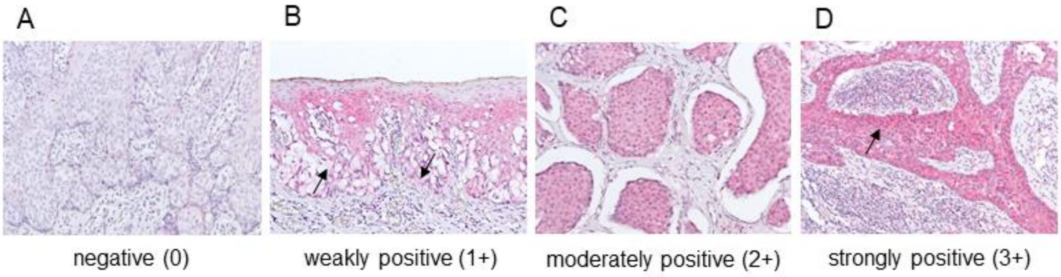

Supplementary Figure S1. Staining intensity of the nectin cell adhesion molecule 4 (NECTIN4) antibody. Positive signals are indicated by red. (A) Negative staining (0), (B) weakly positive staining (1+), (C) moderately positive staining (2+), and (D) strongly positive staining (3+).

Supplementary Figure S2

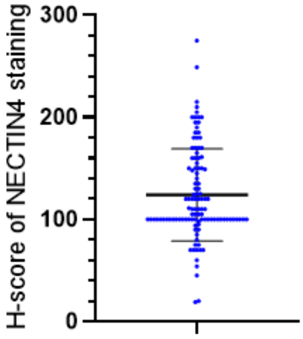

Supplementary Figure S2. H-score for nectin cell adhesion molecule 4 (NECTIN4) staining in extramammary Paget’s disease.
